# Determinants of plasma calretinin in patients with malignant pleural mesothelioma

**DOI:** 10.1186/s13104-020-05187-y

**Published:** 2020-07-29

**Authors:** Martin Lehnert, Daniel G. Weber, Dirk Taeger, Irina Raiko, Jens Kollmeier, Susann Stephan-Falkenau, Thomas Brüning, Georg Johnen, the MoMar-Study Group, Alexander Brik, Katarzyna Burek, Bettina Dumont, Swaantje Casjens, Jan Gleichenhagen, Olaf Hagemeyer, Heike Heimann, Monika Kobek, Claudia Lechtenfeld, Swetlana Meier, Simone Naumann, Christoph Nöllenheidt, Beate Pesch, Simone Putzke, Hans-Peter Rihs, Peter Rozynek, Sandra Schonefeld, Katja Szafranski, Carmen Töpfer, Katharina Wichert, Thorsten Wiethege, Sandra Zilch-Schöneweis

**Affiliations:** 1 Institute for Prevention and Occupational Medicine of the German Social Accident Insurance, Institute of the Ruhr University Bochum (IPA), Bürkle-de-la-Camp-Platz 1, 44789 Bochum, Germany; 2 Clinic for Pneumology, Helios Clinic Emil Von Behring, Walterhöferstr. 11, 14165 Berlin, Germany; 3 Institute for Pathology, Helios Clinic Emil Von Behring, Walterhöferstr. 11, 14165 Berlin, Germany

**Keywords:** Malignant pleural mesothelioma, Molecular marker, Plasma calretinin, Enzyme-linked immunosorbent assay, Tissue staining

## Abstract

**Objective:**

Calretinin is a well-known immunohistochemical tissue marker in the diagnosis of malignant mesothelioma. Promising results also indicate the use in early detection. In the present cross-sectional survey, correlations of calretinin plasma levels with clinical features were investigated.

Plasma samples of 60 patients with malignant pleural mesothelioma (MPM) and 111 cancer-free controls formerly exposed to asbestos were compared. Calretinin concentrations were determined in plasma using an enzyme-linked immunosorbent assay (ELISA).

**Results:**

The median concentration was higher in MPM patients than in controls (0.79 vs. 0.23 ng/ml; *p* < 0.0001). Patients with epithelioid MPM or biphasic MPM had higher calretinin plasma levels than patients with sarcomatoid MPM. Strong expression of calretinin in the tumor tissue was associated with higher plasma levels. Preoperative patients showed higher levels of calretinin than patients after thoracic surgery (1.20 vs. 0.67 ng/ml; *p *= 0.096). The suitability of plasma calretinin has been confirmed as a tumor marker in the differential diagnosis of epithelioid MPM. The value of plasma calretinin for therapy monitoring or as a prognostic marker should be further investigated.

## Introduction

Malignant pleural mesothelioma (MPM) is attributed to an inhalation of asbestos fibers that may date back decades. For 2020, 1160 incident cases of MPM in German men and 320 cases in German women are predicted [1]. Notably, most of the cases in men are recognized as occupationally caused [2].

Usually, MPM emerge with symptoms like shortness of breath or thoracic pain. The cancer is commonly diagnosed at late stages of the disease, resulting in poor survival. Thus, more than half of all patients are going to die within the first year [3]. It is indicated, that a diagnosis at early stages may improve prognosis and treatment options, particularly in view of several promising approaches regarding treatment in numerous clinical trials [4]. Hence, there is a demand for noninvasive markers for the early detection of MPM in at-risk populations.

Today, Calretinin is well established in immunohistochemical marker panels for the diagnosis of MPM [5]. It is a pathfinder in the differentiation of epithelioid MPM and adenocarcinoma of the lung or serous papillary carcinoma of the ovary [6, 7]. A meta-analysis proves the benefit of calretinin determination in pleural effusion for the differentiation of malignant mesotheliomas from other malignancies [8]. Additionally, calretinin might be useful as circulating marker in plasma [9–12]. It is indicated that calretinin might be applicable as marker in field studies, because plasma calretinin is stable regarding repeated freeze/thaw cycles and storage at suboptimal temperatures [9]. Recent results showed the application of plasma calretinin in combination with mesothelin as valid markers for the early detection of MPM in an at-risk population [13]. Furthermore, there are promising efforts to use calretinin as a target for therapeutic agents [14].

However, less is known about clinical factors with impact on the concentration of calretinin in plasma. Thus, the aim of this study was to investigate determinants of plasma calretinin in MPM patients.

## Methods

### Study population

Between July 2008 and April 2017, blood samples and clinical information were collected in a pulmonary clinic from 60 patients with diagnosed MPM (Table 1). The patient group comprised 54 male and 6 female patients aged 32–87 years (median 72 years).

**Table 1:** Description of the study population of cases with MPM

	Male [N]	Female [N]	Total [N]	
Patients [N]	54	6	60	
Age in years [median, range]	72 (32–87)	70 (48–77)	72 (32–87)	
Histological subtype				
Epithelioid	41	6	47	(77.3%)
Sarkomatoid	6	0	6	(10%)
Biphasic	6	0	6	(10%)
Miscellaneous	1	0	1	(1.7%)
Tumor stage	54	6	60	
T1	8	1	9	(26%)
T2	10	0	10	(29%)
T3	7	0	7	(20%)
T4	8	1	9	(26%)
Missing	21	4	25	

The extent of disease in terms of TNM stages was incompletely documented. Valid T stages were available for 35 cases (58%). Therefore, a clinical expert retrospectively estimated the tumor mass at the time of diagnosis on the basis of computed tomography images and stratified the cases according to large and small tumor size. None of the patients were treated with chemotherapy prior to blood collection. The majority of patients (73%) had already had a surgical intervention on the thorax before the blood was taken. One of these patients had also received postoperative radiotherapy a few days before the date of sample collection.

The control group comprised 111 cancer-free individuals of the MoMar cohort with a history of substantial occupational exposure to asbestos and benign asbestos-related diseases (asbestosis, pleural plaques, pleural thickening, and/or pleural fibrosis) [13]. The controls were matched according to age, gender, and smoking status. Each of 54 MPM patients was matched to two controls. For three patients only one control per case could be matched and three cases remained without any match.

All subjects had given written informed consent for enrollment to the MoMar study, which was approved by the ethics committee of the Ruhr University Bochum (reference number 3217-08).

### Blood collection

Blood samples were collected into 9.0 mL S-Monovettes EDTA gel tubes (Sarstedt, Nümbrecht, Germany). The plasma was frozen at − 20 °C immediately after separation by centrifugation (2000 × *g* for 10 min at room temperature within 30 min after collection) and stored temporarily at the cooperating study centers. During transport to the central laboratory using mobile freezing boxes (Dometic Coolfreeze CFX 65 W; Dometic, Emsdetten, Germany) the samples remained frozen at − 20 °C. There the samples were thawed at room temperature for aliquoting and subsequently aliquots were frozen at − 80 °C until use.

### Determination of calretinin

Concentrations of calretinin in plasma samples were determined by enzyme-linked immunosorbent assays (ELISA), using the calretinin ELISA kit by DLD Diagnostika GmbH (Hamburg, Germany; catalog number EA611/96) according to the manufacturer’s instructions. The sensitivity of the ELISA is 0.05 ng/mL, showing a good linearity between 0.25 and 4 ng/mL. The assay is based on the same antibodies as used in the previously described version of the calretinin ELISA [9, 12]. During the procedure, all reagents and samples were equilibrated to 22 °C and the incubations were performed at 22 °C. Plasma samples (30 µL) were diluted 1:5 in the provided dilution buffer. The diluted samples were determined in duplicate. Plasma concentrations are given in ng/mL.

### Immunohistochemistry

Pleural specimens were collected per biopsy or as part of the surgical procedure. Immuno-histochemical staining applying calretinin antibodies in routine procedures was semi-quantitatively assessed with the aim of quantifying the immunoreaction. An H-score was calculated by multiplying the staining intensity by proportion of stained tumor cells. In brief, the staining intensity was assigned to three grades (1: weak, 2: moderate, 3: strong) and proportion of stained cells was stratified into three categories (category 0.1: 0–5%; Category 0.5: > 5–50%; Category 1: > 50–100%). The H-score was calculated to separate calretinin-positive (H-score = 3) from calretinin-negative (H-score < 3) samples based on the median H-score as cut-off [15].

### Statistical analysis

Calretinin plasma concentrations were presented as box plots with median and interquartile range (IQR). Whiskers represent minimum and maximum. One sample showed a calretinin concentration below the limit of detection (LOD) and was therefore set to the LOD (0.005 ng/mL). Non-parametric Mann Whitney's tests were used for group comparisons. Statistics and graphs were performed with GraphPad Prism version 7.04 (GraphPad Software, Inc., San Diego, CA, USA). P values below 0.05 were considered to be statistically significant.

## Results

**Fig. 1:**
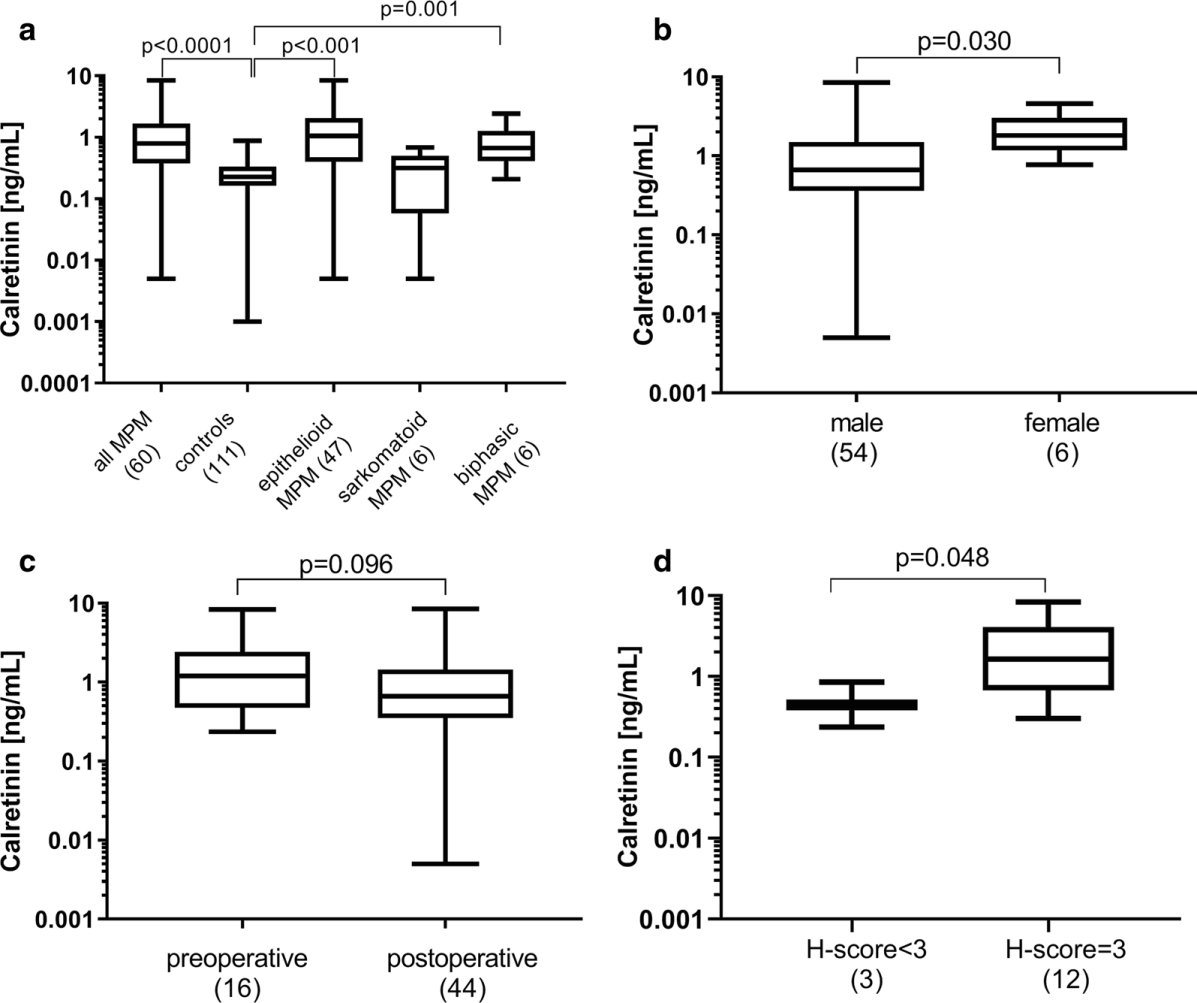
Comparison of calretinin plasma levels in patients with malignant pleural mesothelioma (MPM), matched controls, epithelioid MPM, sarcomatoid MPM, and biphasic MPM (**a**); Comparison of calretinin plasma levels in male and female MPM patients (**b**); Comparison of calretinin plasma levels in pre- and postoperative samples (**c**); Comparison of calretinin plasma levels in patients with high staining response (H-score = 3) and low or moderate staining response (H-score < 3) of tumor tissue in preoperative samples only (**d**)

The median calretinin concentrations in MPM patients and in cancer-free controls were 0.79 ng/mL (IQR 0.37–1.68 ng/mL) and 0.23 ng/mL (IQR 0.16–0.33 ng/mL), respectively (Fig. 1a). The group difference was statistically significant (*p* < 0.0001). The median calretinin concentrations in epithelioid MPM (1.06 ng/mL, IQR 0.40–2.07 ng/mL) and in biphasic MPM (0.67 ng/mL, IQR 0.41–1.28 ng/mL) were significantly higher than the median calretinin concentration in non-diseased controls. Sarcomatoid MPM showed calretinin levels (0.32 ng/mL, IQR 0.06–0.50 ng/mL) similar to the controls.

Male MPM patients (0.66 ng/mL (IQR 0.36–1.50 ng/mL) showed significantly lower calretinin levels (*p* = 0.030) than female MPM patients (1.81 ng/mL (IQR) 1, 17–3.03 ng/mL), *p* = 0.032) (Fig. 1b). No correlation was observed between age and calretinin concentration (r_s_ = − 0.144; *p* = 0.27).

Regarding effects of surgical intervention, calretinin was higher in blood samples drawn before any surgical procedure (1.20 ng/mL, IQR 0.47–2.42 ng/mL) in comparison to samples drawn afterwards (0.67 ng/mL, IQR 0.35–1.45 ng/mL), but this group difference was statistically not significant (*p* = 0.096) (Fig. 1c).

H-score was calculated for the 15 preoperative mesothelioma samples (Fig. 1d). The majority of mesothelioma were calretinin-positive (H-score = 3), whereas only three tissue specimens were calretinin-negative (H-score < 3). The difference in plasma levels was just statistically significant (*p* = 0.048).

The stage of disease in terms of T stage by the Union for International Cancer Control (UICC) was recorded for 35 MPM patients. Restricted to epithelioid and biphasic MPM higher medians of calretinin were observed in patients with stage T1 (1.06 ng/mL, IQR 0.64–1.48 ng/mL) and stage T2 (1.34 ng/mL, IQR 0.50–2.18 ng/mL) than in patients with stage T3 (0.45 ng/mL, IQR 0.28–0.82 ng/mL) and stage T4 (0.53 ng/mL, IQR 0.37–4.67 ng/mL).

After restriction to preoperative samples (N = 16), median calretinin levels of 0.81 ng/mL (IQR 0.45–1.80) for patients with a smaller tumor mass and 1.31 ng/mL (IQR 0.88–2.76) for patients with larger tumor mass were observed. The difference was not statistically significant (*p* = 0.23).

## Discussion

Higher plasma calretinin concentrations were observed in patients with epithelioid or biphasic MPM compared to patients with sarcomatoid MPM or cancer-free controls. This is in accordance with previous analyses showing similar results [9, 12].

Higher calretinin concentrations were observed in the six women in comparison to the 56 men. Based on the small number of female participants in the study group, random results could not be excluded, but similar gender-based differences were already observed [10, 11]. Higher calretinin serum levels in women might be based on the expression of calretinin in ovarian tissue [16]. Recently, the value of serum calretinin in the prognosis and treatability of ovarian cancer has been reported [17]. However, due to a higher probability of an occupational exposure to asbestos the risk for MPM is higher in men than in women. In the total population of Germany a gender ratio of 3.8 affected men to one affected woman is observed [2]. Therefore, most of studies focus on male workers who were previously exposed to asbestos. However, to evaluate a gender effect on calretinin the inclusion of higher numbers of female patients with MPM and female controls formerly exposed to asbestos is needed.

To the best of our knowledge, this is the first time that the immunostaining of tumor tissue has been compared with the concentration of circulating calretinin in human plasma.

More intense staining of the tumor tissue, assessed by the H-score, was associated with significantly higher plasma calretinin. Conversely, it was confirmed that low calretinin expression of the tumor correlates to small amounts of the protein released into the bloodstream. In contrast to observations on mesothelin [18], no clear correlation between the size of the primary tumor and the calretinin level in plasma could be confirmed. While the results, based on an expert evaluation of the individual tumor mass, pointed in this direction, there was no trend of an increase in calretinin levels from stage T1 to T4. Instead, T2 patients had the highest median and T3 patients the lowest median calretinin concentration. Since this observation was made in very small numbers of cases within the different T categories, further investigations with higher numbers of patients with the individual T stages are necessary. However, the release of circulating proteins could be controlled by other tumor-specific processes in addition to the tumor mass. For example, cancer cells may facilitate the secretion of invasion-promoting proteins [19] and this may also be true for calretinin, as calretinin has been suggested to promote invasiveness [20].

For mesothelin, serum levels have been reported to decrease after surgery [21]. A similar trend was observed for plasma calretinin, with a tendency to lower calretinin levels in postoperative patients compared to preoperative patients. Generally, surgical reduction of the tumor mass may reduce the concentration of circulating tumor markers. However, tissue manipulation could initially cause a short-term increase of tumor markers in plasma.

## Conclusion

Patients with epithelioid or biphasic MPM showed higher calretinin concentrations than cancer-free controls with benign asbestos-related lung diseases. The intensity of the immune histochemical staining of the tumor tissue was positively associated with the plasma calretinin levels of the patients. A clear association between plasma calretinin and the extent of the disease could not be demonstrated. Trends towards lower calretinin levels after surgical debulking may indicate a benefit in monitoring the efficacy of therapy and disease progression.

## Limitations

A limitation of this study was the small number of patients in the subgroups. Thus, random effects cannot be excluded. Blood samples from 16 patients were taken before thoracic surgery, whereas from 44 patients blood samples were taken after interventions. Although the group differences were not statistically significant, the observed trend is physiologically understandable. To further assess the benefit of plasma calretinin, larger studies with a balanced gender ratio and defined sampling times are needed. Additionally, for the evaluation of plasma calretinin for monitoring samples should be collected prospectively during treatment.

For a more detailed analysis of the relationship between calretinin levels and tumor mass, more detailed information on the disease, e.g., the extent of the primary tumor in the form of a T stage, is required.

## Supplementary information

**Additional file 1: **Table of key variables and measured values.

## Data Availability

A table with key variables from our dataset is available as additional material (Additional file 1). Further material and information is available on request from the corresponding author.

## Abbreviations

ELISA: Enzyme-linked immunosorbent assays
IQR: Interquartile range
LOD: Limit of detection
MPM: Malignant pleural mesothelioma
UICC: Union for International Cancer Control
